# Application of High-Quality Dried Olive with High Polyphenol Content for Bread Fortification: Effects on Nutritional, Technological, and Sensory Properties

**DOI:** 10.3390/molecules30173564

**Published:** 2025-08-30

**Authors:** Jorge Saura-Martínez, Luis Tortosa-Díaz, Francisco José López-Avilés, Miguel Juárez-Marín, Asunción María Hidalgo, Fulgencio Marín-Iniesta

**Affiliations:** 1 Group of Research Food Biotechnology-BTA, Department of Food Science, Nutrition and Bromatology, Regional Campus of International Excellence “Campus Mare Nostrum”, University of Murcia, 30100 Murcia, Spain; j.sauramartinez@um.es (J.S.-M.); luis.tortosad1@um.es (L.T.-D.); franciscojose.lopez4@um.es (F.J.L.-A.); miguel.juarez@um.es (M.J.-M.); 2 Department of Chemical Engineering, Regional Campus of International Excellence “Campus Mare Nostrum”, University of Murcia, 30100 Murcia, Spain; ahidalgo@um.es

**Keywords:** hydroxytyrosol, waste management, shelf life, circular economy, nutraceutics, dietary fibre

## Abstract

New types of fortified breads have been developed from a new ingredient obtained by a two-step process for olive oil extraction, without external water addition, called high-quality dried olive (HQDO). HQDO is a minimally processed ingredient rich in phenolic compounds with beneficial health properties. HQDO has been incorporated in different percentages (1% HDQO1, 5% HDQO5, 10% HDQO10, and control CON) to study how it affects the properties of bread. The effects on the texture and shelf-life extension of the breads have also been studied. The bread samples were evaluated by a trained panel in descriptive sensorial analysis (1–10 scale). Fortified breads improve their nutraceutical quality by increasing their phenolic content from 0.19 mg GAE g^−1^ CON to 0.73 mg GAE g^−1^ at HDQO10 (using the Folin–Ciocalteu method). Antioxidant activity was increased from 1.24 mg AAE g^−1^ CON to 1.49 mg AAE g^−1^ HDQO10 (using the DPPH method). In sensory properties, all fortified breads obtained a high punctuation with a rating near to seven or superior. In “Aroma” and “Aftertaste”, the fortified breads obtained superior ratings. Finally, in “Flavour”, except for HQDO10, all of them had values close to eight. HDQO1 and HDQO5 were selected for their equilibrium between nutritional qualities and sensorial evaluation.

## 1. Introduction

The search for foods with health benefits is a growing trend. The importance of diet in well-being and disease prevention is leading to changes in consumer habits and in the food industry to meet market needs. There is also a trend towards more sustainable food production, making the most of agri-food by-products.

Olive oil, especially extra virgin olive oil (EVOO), is recommended for its numerous health benefits, mainly due to its high content of antioxidants and healthy fatty acids [1]. The production of olive oil, the main product derived from olives, generates a large amount of by-products during the process. The most used olive oil extraction process is called two-phase extraction [2]. This two-phase process generates a large amount of a by-product known as olive pomace [3]. Olive pomace is composed of the solid fraction (remnants of pulp, skin, and pits) and the aqueous fraction of the olive (water content, with dissolved water-soluble compounds) [4,5]. This by-product accounts for between 70 and 80% of the total weight of fresh olives. Olive pomace is usually stored in open-air ponds to reduce its water content. It is then used to obtain olive pomace oil, animal feed, compost, or biomass for burning. This represents an underutilization of a by-product rich in bioactive compounds associated with numerous environmental problems [4].

However, both fractions can be separated right after obtaining the olive oil through a process of centrifugation and drying under hygienic conditions, avoiding oxidations and fermentations [6]. Centrifugation separates the aqueous fraction, and the solid part is dried with hot air to obtain high-quality dried olive (HQDO), which is ground to obtain flour. This way, a high-quality product can be obtained in a zero-waste process. HQDO is rich in a wide variety of phenolic compounds with bioactive effects present in olive oil (hydroxytyrosol, tyrosol, caffeic acid, *p*-coumaric acid, vanillic acid, syringic acid, gallic acid, luteolin, quercetin, cyanidin, and verbascoside, among others). Hydroxytyrosol is the main bioactive compound in olives. This compound is recognised by the European Food Safety Authority (EFSA) as having the ability to promote a beneficial effect on human health when consumed regularly, and a minimum daily intake of 5 mg is recommended [7]. Furthermore, their concentration in phenolic compounds can be 30 to 40 times higher than their concentration in olive oil, due to their water-soluble nature. HQDO is also rich in dietary fibre and a reduced fatty acid content.

There is ample evidence of the positive effect of olives on human health [1,8] and the growing consumer trend towards healthy eating [9]. Fortifying bakery products with olive by-products seems to be an interesting strategy for enriching their nutritional properties [10,11,12,13,14,15,16,17].

The aim of this research is, therefore, to develop new fortified bread recipes with HQDO to improve its nutritional, sensory, and technological properties compared to conventional bread.

## 2. Results

### 2.1. Crust and Crumb Colourimetry

**Figure 1:**
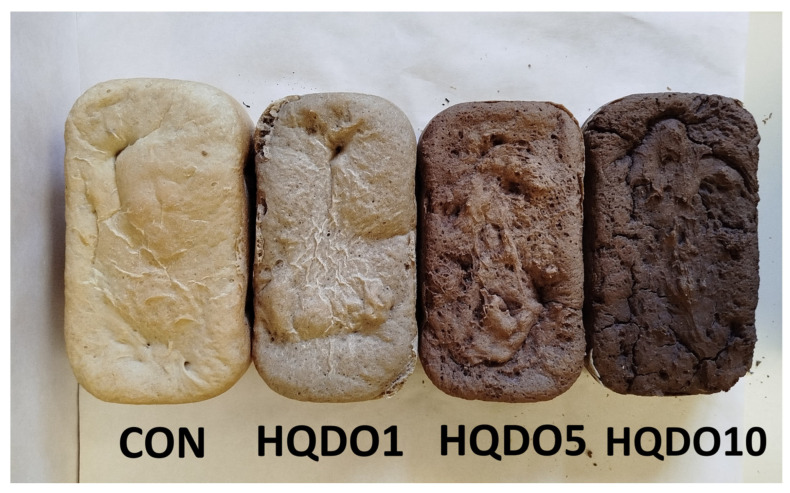
Bread crust colour comparison of the four bread samples. CON: unfortified bread (control); HQDO1: 1% flour substitution; HQDO5: 5% flour substitution; HQDO10: 10% flour substitution. HQDO: high-quality dried olive.

**Figure 2:**
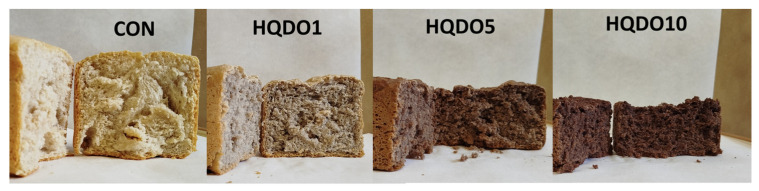
Bread crumb colour comparison of the four bread samples. CON: unfortified bread (control); HQDO1: 1% flour substitution; HQDO5: 5% flour substitution; HQDO10: 10% flour substitution. HQDO: high-quality dried olive.

The effects of HQDO fortification on the colour of bread crust and crumb are shown in Table 1 and Table 2 and Figure 1 and Figure 2.

The addition of HQDO significantly affected the colour of the crust and crumb. It was observed that both L* and b* decreased proportionally with increasing HQDO content in both the crust and crumb. In Figure 1, we saw a large difference in the colour of bread crust. This difference is reflected in Table 1, especially in the lightness value (L*), which ranges from 64.3 in CON to 30.6 in HQDO10 (52.4% decrease). In the bread crumb, it was observed that the L* value ranges from 56.0 in CON to 28.7 in HQDO10, showing a similar decrease (48.7% decrease).

Similarly, a* and BI increased, following the same trend. BI increased due to the appearance of Maillard compounds from the baking process and colouring compounds present in HQDO. hº decreased in both the crust and the crumb as the HQDO concentration was increased. Finally, TCD increased in both parts as the HQDO concentration in the bread was increased. The observed trend of a* and b* indicates a variation to a browner colouring.

### 2.2. Height and Weight Loss

**Figure 3:**
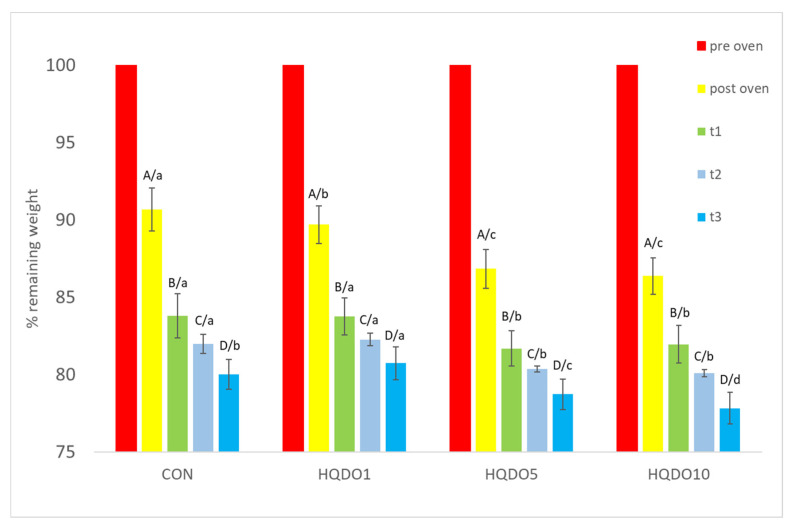
Remaining weight of bread in the four bread samples during 0, 1, 2, and 3 days. CON: unfortified bread (control); HQDO1: 1% flour substitution; HQDO5: 5% flour substitution; HQDO10: 10% flour substitution. HQDO: high-quality dried olive. Each variable that shares the same letter shares a mean that is not statistically different from another (*p* < 0.05). Lowercase letters (a, b, c, and d) relate data between different types of bread at the same time value. Uppercase letters (A, B, C, and D) relate data of the same type of bread at different time values.

Weight loss comparison of the four breads tested is shown in Figure 3.

To analyse weight loss, two types of measures were used: weight post-oven and weight during shelf life. Increasing the amount of HQDO in the formula increased weight loss both after baking and over time. While CON went from 90.64% remaining post-oven to 80% remaining after 3 days, breads with HQDO showed a greater increase (HQDO1 from 89.68% remaining post-oven to 80.72% remaining after 3 days, HQDO5 from 86.82% remaining post-oven to 78.72% remaining after 3 days, and HQDO10 from 86.36% remaining to 77.81% remaining after 3 days).

**Figure 4:**
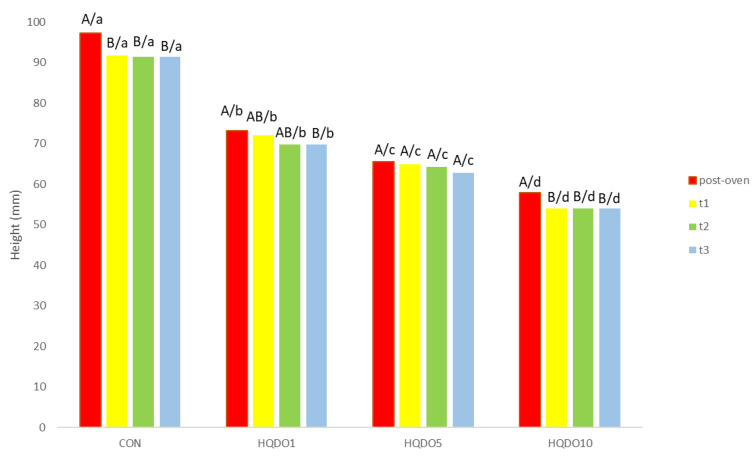
Height of bread in the four bread samples during 0, 1, 2, and 3 days. CON: unfortified bread (control); HQDO1: 1% flour substitution; HQDO5: 5% flour substitution; HQDO10: 10% flour substitution. HQDO: high-quality dried olive. Each variable that shares the same letter shares a mean that is not statistically different from another (*p* < 0.05). Lowercase letters (a, b, c, and d) relate data between different types of bread at the same time value. Uppercase letters (A, B, and AB) relate data of the same type of bread at different time values.

**Figure 5:**
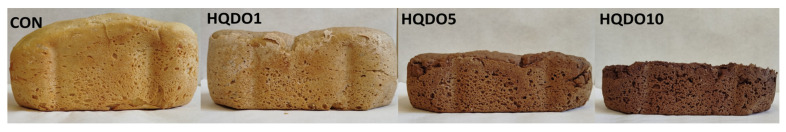
Visual bread height comparison in the four bread samples at t0. CON: unfortified bread (control); HQDO1: 1% flour substitution; HQDO5: 5% flour substitution; HQDO10: 10% flour substitution. HQDO: high-quality dried olive.

Height loss comparisons of the four breads tested are shown in Figure 4 and Figure 5.

**Figure 6:**
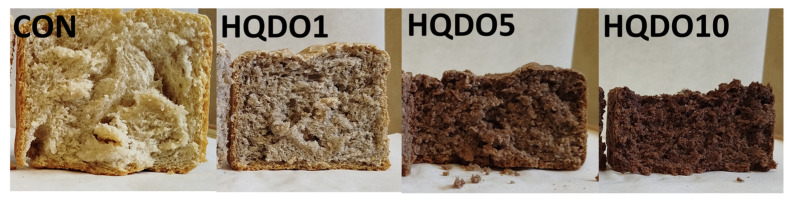
Visual bread crumb comparison in the four bread samples at t0. CON: unfortified bread (control); HQDO1: 1% flour substitution; HQDO5: 5% flour substitution; HQDO10: 10% flour substitution. HQDO: high-quality dried olive.

In terms of height, the higher the concentration of HQDO in bread was, the lower the bread height. A more compact and less porous crumb with small air cells was observed, as shown in Figure 6.

While CON went from 97.25 mm post-oven to 91.5 mm after 3 days, the breads with HQDO show a greater reduction (HQDO1 from 73.25 mm post-oven to 69.75 mm after 3 days, HQDO5 from 65.5 mm post-oven to 62.75 mm after 3 days, and HQDO10 from 58 mm post-oven to 54 mm after 3 days).

### 2.3. Phenolic Compounds and Antioxidant Capacity Analyses

**Figure 7:**
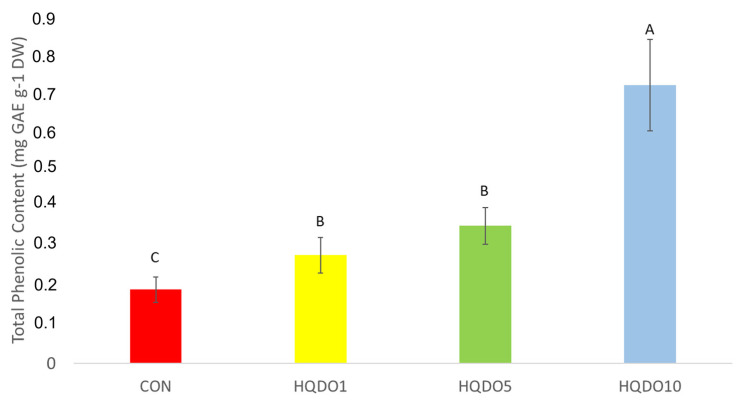
Total phenolic content in the four bread samples. CON: unfortified bread (control); HQDO1: 1% flour substitution; HQDO5: 5% flour substitution; HQDO10: 10% flour substitution. HQDO: high-quality dried olive. Each variable that shares the same letter shares a mean that is not statistically different from another. (*p* < 0.05).

Total phenolic content comparison of the four breads tested is shown in Figure 7.

It was observed that phenolic compound concentration increases with the increase in HQDO content in the recipe. While CON had 0.19 mg GAE g^−1^ DW, in breads with HQDO, the polyphenol concentration increased as the concentration of HQDO increased (0.28 mg GAE g^−1^ DW HQDO1, 0.36 mg GAE g^−1^ DW HQDO5, and 0.73 mg GAE g^−1^ DW HQDO10).

**Figure 8:**
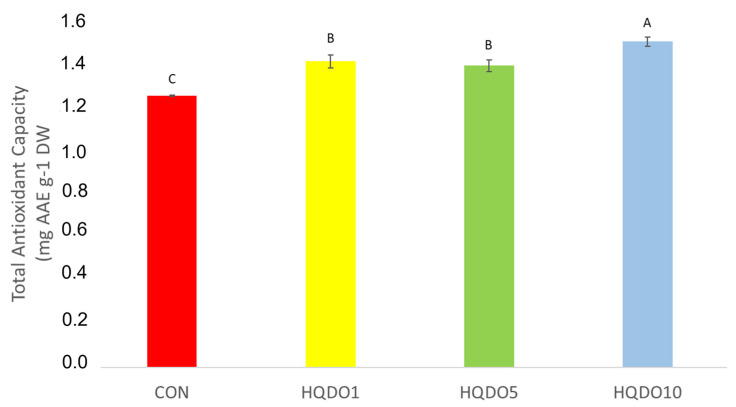
Antioxidant capacity in the four bread samples. CON: unfortified bread (control); HQDO1: 1% flour substitution; HQDO5: 5% flour substitution; HQDO10: 10% flour substitution. HQDO: high-quality dried olive. Each variable that shares the same letter shares a mean that is not statistically different from another (*p* < 0.05).

Total antioxidant capacity comparison of the four breads tested can be seen in Figure 8.

It was observed that antioxidant capacity increases with the increase in HQDO content in the recipe. While CON had 1.24 mg AAE g^−1^ DW, breads with HQDO show an increase in polyphenol concentration as the concentration of HQDO increased (1.40 mg AAE g^−1^ DW HQDO1, 1.38 mg AAE g^−1^ DW HQDO5, and 1.49 mg AAE g^−1^ DW HQDO10).

### 2.4. Texture Evolution

**Figure 9:**
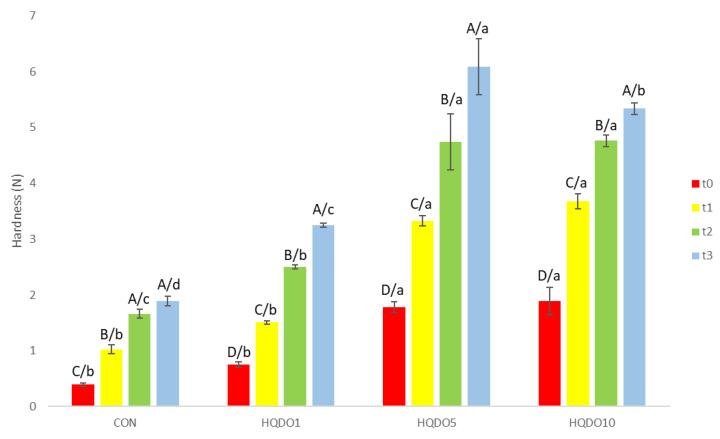
Hardness evolution in the four bread samples during 0, 1, 2, and 3 days. CON: unfortified bread (control); HQDO1: 1% flour substitution; HQDO5: 5% flour substitution; HQDO10: 10% flour substitution. HQDO: high-quality dried olive. Each variable that shares the same letter shares a mean that is not statistically different from another (*p* < 0.05). Lowercase letters (a, b, c, and d) relate data between different types of bread at the same time value. Uppercase letters (A, B, C, and D) relate data of the same type of bread at different time values.

Hardness evolution during the 4-day trial of the four breads tested is shown in Figure 9.

Crumb hardness increased with higher concentrations of HQDO in bread. With respect to time, the crumb of breads with a higher concentration of HQDO increased in hardness more quickly. While CON increased from 0.39 N to 1.89 N, breads with HQDO showed a greater increase (HQDO1 from 0.75 N to 3.24 N, HQDO5 from 1.78 N to 6.09 N, and HQDO10 from 1.89 N to 5.34 N).

**Figure 10:**
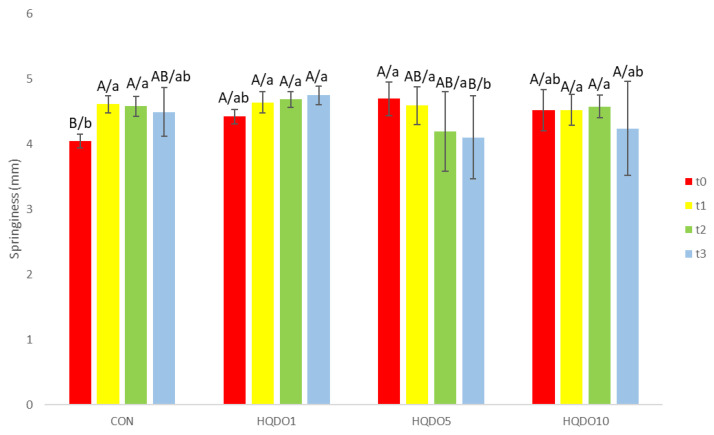
Springiness evolution in the four bread samples during 0, 1, 2, and 3 days. CON: unfortified bread (control); HQDO1: 1% flour substitution; HQDO5: 5% flour substitution; HQDO10: 10% flour substitution. HQDO: high-quality dried olive. Each variable that shares the same letter shares a mean that is not statistically different from another (*p* < 0.05). Lowercase letters (a, b, and ab) relate data between different types of bread at the same time value. Uppercase letters (A, B, and AB) relate data of the same type of bread at different time values.

Springiness evolution during the 4-day trial of the four breads tested is shown in Figure 10.

We observed that adding HQDO to the dough at different concentrations has a slight effect on the dough’s springiness. On the other hand, more evident differences were observed in the variation in the dough’s springiness over time. As the concentration of HQDO increased, the springiness of the dough decreased compared to the CON bread, where an increase in springiness was observed over the 4 days (CON from 4.04 mm to 4.48 mm, HQDO1 from 4.41 mm to 4.74 mm, HQDO5 from 4.69 mm to 4.1 mm, and HQDO10 from 4.52 mm to 4.23 mm).

**Figure 11:**
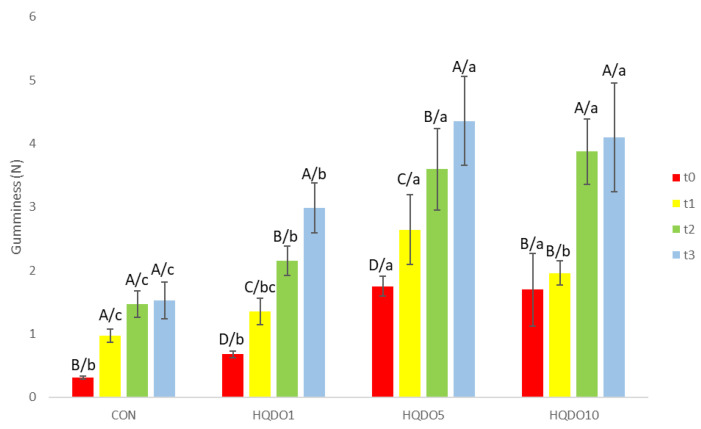
Gumminess evolution in the four bread samples during 0, 1, 2, and 3 days. CON: unfortified bread (control); HQDO1: 1% flour substitution; HQDO5: 5% flour substitution; HQDO10: 10% flour substitution. HQDO: high-quality dried olive. Each variable that shares the same letter shares a mean that is not statistically different from another (*p* < 0.05). Lowercase letters (a, b, and bc) relate data between different types of bread at the same time value. Uppercase letters (A, B, C, and D) relate data of the same type of bread at different time values.

Gumminess evolution during the 4-day trial of the four breads tested is shown in Figure 11.

Crumb gumminess increased with higher concentrations of HQDO in bread. With respect to time, the crumb of breads with a higher concentration of HQDO increased in gumminess more quickly. While CON went from 0.31 N to 1.53 N, breads with HQDO showed a greater increase (HQDO1 from 0.68 N to 2.98 N, HQDO5 from 1.75 N to 4.35 N, and HQDO10 from 1.69 N to 4.1 N).

**Figure 12:**
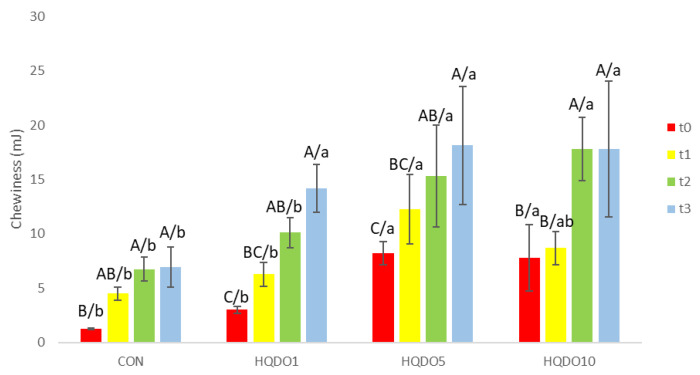
Chewiness evolution in the four bread samples during 0, 1, 2, and 3 days. CON: unfortified bread (control); HQDO1: 1% flour substitution; HQDO5: 5% flour substitution; HQDO10: 10% flour substitution. HQDO: high-quality dried olive. Each variable that shares the same letter shares a mean that is not statistically different from another (*p* < 0.05). Lowercase letters (a, b, and ab) relate data between different types of bread at the same time value. Uppercase letters (A, B, AB, BC, and C) relate data of the same type of bread at different time values.

Chewiness evolution during the 4-day trial of the four breads tested is shown in Figure 12.

Crumb chewiness increased with higher concentrations of HQDO in bread. With respect to time, the crumb of breads with a higher concentration of HQDO increased in chewiness more quickly. While CON went from 1.26 mJ to 6.93 mJ, breads with HQDO show a greater increase (HQDO1 from 3 mJ to 14.16 mJ, HQDO5 from 8.23 mJ to 18.13 mJ, and HQDO10 from 7.8 mJ to 17.8 mJ).

### 2.5. Accelerated Mould Growth Inhibition Test

**Figure 13:**
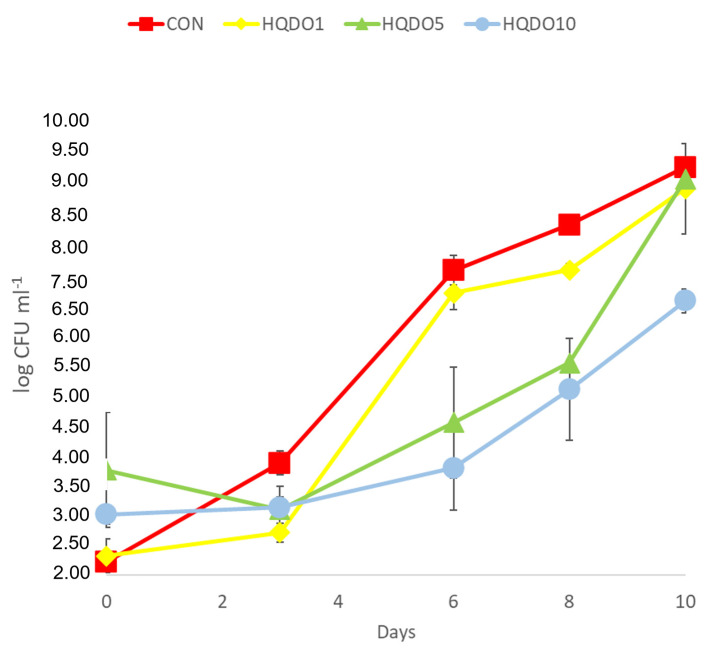
Evolution of mould and yeast counts in the accelerated mould growth inhibition test for the four types of bread over 10 days. CON: unfortified bread (control); HQDO1: 1% flour substitution; HQDO5: 5% flour substitution; HQDO10: 10% flour substitution. HQDO: high-quality dried olive.

Mould and yeast growth during the 10-day trial of the four breads tested is shown in Figure 13.

An initial count of less than 2.5 log CFU g^−1^ was observed, with no differences between CON and HQDO1. In the case of HQDO5 and HQDO10, a higher count was observed (3.75 log CFU g^−1^ and 3.01 log CFU g^−1^, respectively). This may be due to the native yeast flora present in HQDO that survives the initial treatment, drying, and baking. All HQDO breads showed mould inhibition until day 3, with differences (2.71 log CFU g^−1^ HQDO1, 3.09 log CFU g^−1^ HQDO5, and 3.12 log CFU g^−1^ HQDO10) compared to the control sample (3.86 log CFU g^−1^), where mould growth was observed. From day 6 onwards, moulds appear in HQDO1, reflecting greater growth (6.69 log CFU g^−1^), while HQDO5 and HQDO10 maintained lower counts (4.54 log CFU g^−1^ HQDO5 and 3.78 log CFU g^−1^ HQDO10). Until day 8, mould growth was similar between HQDO5 and HQDO10, with differences (7.07 log CFU g^−1^ HQDO1, 5.53 log CFU g^−1^ HQDO5, and 5.09 log CFU g^−1^ HQDO10) compared to CON (7.82 log CFU g^−1^). At day 10 counts, only the HQDO10 sample had shown lower counts (6.56 log CFU g^−1^ HQDO10) than the other samples (8.78 log CFU g^−1^ CON, 8.43 log CFU g^−1^ HQDO1, and 8.59 log CFU g^−1^ HQDO5).

### 2.6. Descriptive Sensorial Analysis

**Figure 14:**
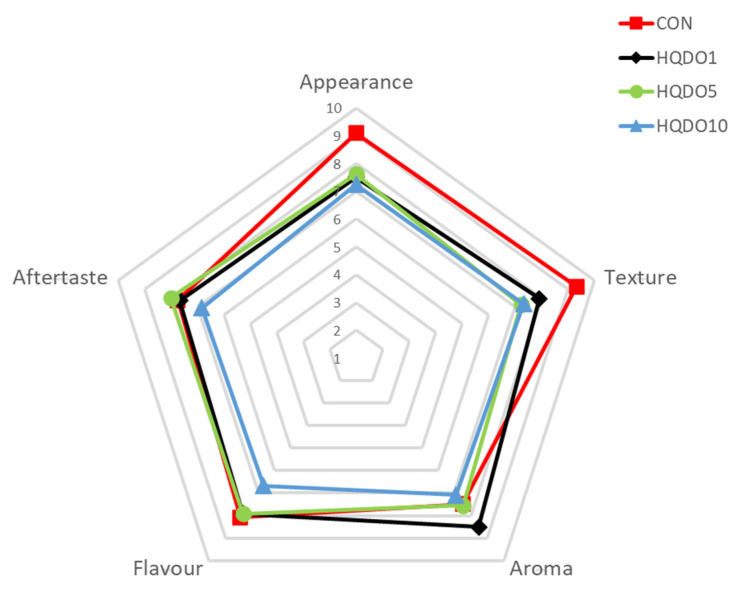
Sensory evaluation of the four bread samples. CON: unfortified bread (control); HQDO1: 1% flour substitution; HQDO5: 5% flour substitution; HQDO10: 10% flour substitution. HQDO: high-quality dried olive.

Sensory evaluation results of the four breads tested are shown in Figure 14 and Table 3.

It was observed that the increasing incorporation of HQDO has a strong sensory impact on bread. The attribute most affected was colour, as discussed in Section 3.1. The other attributes most affected by the increasing incorporation of HQDO were “Appearance” and “Texture”. In terms of “Appearance”, CON obtained the best values, while the other breads obtained slightly lower values that were very similar to each other. In contrast, for “Texture”, CON obtained the best result (9.3), followed by HQDO1 (7.9) (*p* < 0.05). HQDO5 and HQDO10 obtained lower values (7.3 and 7.2). This is interesting, given that the differences observed in the parameters studied in Section 3.6 between HQDO5 and HQDO10 bread were not reflected in sensory perception (*p* < 0.05). In terms of “Aroma”, HQDO1 bread was the highest rated (8.5), while the other three breads had similar and slightly lower values (CON 7.5, HQDO5 7.5 and HQDO10 7.1) (*p* < 0.05). In terms of “Flavour”, HQDO10 was the lowest rated (6.7). The other breads showed a slightly higher value (CON 8.1, HQDO1 7.9, and HQDO5 7.9) (*p* < 0.05), as the fortifying compound has a significant bitter taste that may not be to the liking of all consumers. Finally, the same trend was observed in the case of aftertaste. HQDO10 was the lowest rated (6.8). The other breads had a slightly higher value (CON 7.8, HQDO1 7.7, and HQDO5 8).

## 3. Discussion

### 3.1. Crust and Crumb Colourimetry

Regarding colourimetry results, as shown in Table 1 and Table 2, there was a clear relationship between the colour intensity of bread crust and crumb and the concentration of HQDO. In both cases, a change in colour was observed, with the most notable being the decrease in the L* coordinate value (lightness), while the a * coordinates showed an increase. However, the b * coordinates tend to decrease. As BI increased, WI decreased, indicating increasingly darker pigmentation.

Dahdah et al. [14] made bread with olive pomace from two different olive cultivars, Bosana and Semidana, which had a yellowish colour. When analysing crust colour, L* increased in both cases (Bosana to 65.6 at 1% and Semidana to 69.9 at 1%, compared to the control bread with 43.9). On the other hand, a* (Bosana up to −1.3 at 1% and Semidana to −1.1 at 1%, compared to the control bread with 5.6) and b* (Bosana up to 6.9 at 1% and Semidana to 8.5 at 1%, compared to the control bread with 14.2) decreased. The BI also decreased (Bosana to 34.4 at 1% and Semidana to 30.1 at 1%, compared to the control bread with 56.1). As for crumb, L* decreased in both cases (Bosana to 62.2 at 1% and Semidana to 63.9 at 1%, compared to the control bread with 72.3). On the other hand, a* increased (Bosana to −1.2 at 1% and Semidana to −1.4 at 1%, compared to the control bread with −1.9), while b* decreased (Bosana to 11.5 at 1% and Semidana to 11.4 at 1%, compared to the control bread with 12.7). The WI also decreased (Bosana to 60.5 at 1% and Semidana to 62.1 at 1%, compared to the control bread with 69.4).

Black olives are fruits with a high pigment content. Specifically, the Arbequina cultivar has an intense dark colour due to its high content of anthocyanins, carotenoids, chlorophylls, and xanthines [18,19]. These pigments are also present in HQDO, which contributes to bread colour. The Bosana and Semidana cultivars have a different colouring than the Arbequina cultivar used in this study. Another parameter that influences colour is the ripening time of the fruit before processing, as the concentration of pigments varies throughout the fruit’s development.

The colour of the crust can be affected by the fortifying compound, Maillard reactions, and caramelisation. These types of reactions also depend on pH, temperature, and water activity [20]. Maillard reactions occur between the amino groups of proteins and reducing sugars, such as fructose. These reactions occur when products are exposed to high temperatures and affect the colour, flavour, aroma, and texture of the final product [15]. When sugars condense at the end of the reaction, melanoidins are formed, giving the product a brown colour [15,20].

### 3.2. Height and Weight Loss

In terms of weight and height loss, our results were similar to other studies with fortified breads. This trend was also observed by Singh et al. [21], who made cake batter using a fibre-rich ingredient, corn bran, and found that weight loss increased with the amount added, with a 9.41% loss of moisture when 10% corn bran was added compared to the control bread, which had a 9.04% loss.

Kasprzak, M., and Rzedzicki, Z. [22], demonstrated that the higher the concentration of pea husks in the dough, the lower the oven loss. While the control bread had a weight loss of 13.62% per bake, adding 10% small-grain pea husks and large-grain pea husks reduced this to 12.49% and 12.75%, respectively. Regarding height loss, it was shown that the higher the concentration of pea seed coat in the dough, the lower the increase in bread volume. While the control bread had a volume of 385 cm^3^ 100 g^−1^, adding 10% small-grained pea hulls and coarse-grained pea hulls reduced the volume to 257 cm^3^ 100 g^−1^ and 247 cm^3^ 100 g^−1^, respectively. The breads fortified with pea seed coat also showed lower crumb porosity (49.98% small-grained pea hulls and 51.85% coarse-grained pea hulls) and alveolar size compared to the control bread (71.37%).

Total baking loss depends on many factors, such as the shape, size, volume, and type of bread, the method of dough management and baking, and even the type of oven used [22].

Although HQDO has a high concentration of dietary fibre (48.0 ± 17 g 100 g^−1^), which is capable of absorbing and retaining water, it also has a high concentration of fatty acids (17.2 ± 1.7 g 100 g^−1^), which can prevent this absorption and allow it to diffuse more quickly to the outside.

Dahdah et al. [14] incorporated olive pomace from the Bosana and Semidana olive cultivars in different proportions into the bread dough. In both cases, there was a decrease in bread height and an increase in the number of air cells (193.52 cells/cm^2^ Bos 1% and 188.48 cells/cm^2^ Sem 1%) compared to the control bread (147.19 cells/cm^2^).

Bread structure consists of a fluid phase (air) and a solid phase. This solid phase is composed of intertwined starch and gluten molecules together with other components to form a three-dimensional network with viscoelastic properties [23,24]. Throughout bread’s shelf life, the water contained in the mixture diffuses from the inside to the outside, causing it to lose moisture and interact with the different fractions of the bread. This process changes the bread properties due to the interaction of water with gluten and starch polymers, which have different water retention capacities. Water retention is very important throughout bread’s shelf life, as it determines crust hardness and crumb springiness, among other characteristics.

Replacing flour with HQDO hinders the formation of the three-dimensional network between gluten and starch and removes substrate for yeast fermentation, reducing gas and air cell formation in dough. The presence of antioxidant and antimicrobial compounds in HQDO can also affect yeast metabolism, slowing it down and reducing dough growth. These factors can influence the rise of the dough during the baking process. The height and volume of bread are important parameters for evaluating its market value, as they are associated with product quality at the consumer level [25]. Determining the fortification effect on the final product is important to ensure consumer acceptance of the product.

### 3.3. Phenolic Compounds and Antioxidant Capacity Analyses

In terms of TPC and antioxidant capacity, these results were similar to those of other studies.

Dahdah et al. [14] incorporated olive pomace from the Bosana and Semidana olive cultivars in different proportions into the bread dough. In both cases, the antioxidant capacity of the bread increased due to the compounds present in the olive by-products. In the case of Bosana, it increased to 32.9 μmol TE g^−1^ DW at 1%, compared to the control bread with 21.4 μmol TE g^−1^ DW, while with Semidana, it increased to 39.9 μmol TE g^−1^ DW at 1%.

Cedola et al. [12] incorporated 10% olive pomace of the Cellina di Nardò cultivar to test its effect on bread nutritional quality. The fortified bread had more total phenols (45.09 mg GAE g^−1^ DW) than the control bread (0.29 mg GAE g^−1^ DW). In terms of antioxidant capacity, the fortified bread showed more antioxidant capacity (21.64 mg Trolox equivalent g^−1^ DW) than the control bread (0.24 mg Trolox equivalent g^−1^ DW).

Cardinali et al. [10] incorporated olive pomace of the Piantone di Mogliano cultivar processed using cold treatments in different proportions to test its effect on the nutritional quality of bread. In all cases, the incorporation of olive pomace increased both the TPC and the antioxidant capacity.

Although an increase was seen in all cases when the product was incorporated into bread, different rates of increase were observed. These differences may be due to the olive cultivar used, flour type, and the baking process. There are Maillard reaction products that also have antioxidant activity, so baking characteristics may affect these measurements [14]. Another important factor is the sensitivity of olive polyphenols to temperature. Processes that require an increase in temperature can drastically diminish the concentration of polyphenols in olive oil, reducing its quality [26,27].

It is well established that olive by-products contain high concentrations of phenolic compounds with antioxidant properties. These compounds have beneficial health properties, such as anti-inflammatory, anti-carcinogenic, cardioprotective, etc. [1,8]. The incorporation of these healthy compounds into foods that are widely consumed by the population can help to correct or prevent widespread nutritional deficiencies, with the aim of balancing the overall nutritional profile of diets [28]. The fortification of bakery products had been studied in numerous studies with both olive by-products [10,11,12,13,14,15,16] and other plant by-products [17,21,22,29,30].

### 3.4. Texture Evolution

Our results were similar to those of Dahdah et al. [14], who observed that increasing the olive pomace content of two olive cultivars significantly increased hardness compared to the control bread (11.78 N), with the increase being greater in the 1% olive pomace of the Bosana cultivar (25.43 N) than in the 1% olive pomace of the Semidana cultivar (25.14 N).

In the case of springiness, they observed that the increase in olive pomace from both cultivars does not produce changes in the dough.

They observed that increasing the olive pomace content of two olive cultivars significantly increases gumminess compared to the control bread (10.58 N), with the Bosana cultivar at 1% (22.12 N) showing greater gumminess than the Semidana cultivar at 1% (21.55 N).

In the case of chewiness, they observed that increasing the olive pomace content of two olive cultivars significantly increased chewiness compared to the control bread (10.63 mJ), with the Bosana cultivar at 1% (32.52 mJ) showing greater chewiness than the Semidana cultivar at 1% (27.07 mJ).

The rheological properties of bread dough are influenced by the formation of the three-dimensional network. The proportion of gluten, starch, and other components determines how it behaves structurally. The incorporation of HQDO reduces the amount of gluten and starch in the dough by substitution, as well as providing a large amount of fibre. Water retention capacity also has an influence. As described previously in the study, increasing HQDO concentration reduces water retention, causing it to harden sooner. Enriching bread with alternative ingredients generally decreases the sensory quality of the bread because it alters the formation of networks and destabilises the gas cells, causing low gas retention [12]. This effect was more evident when higher concentrations of HQDO were added to the dough, resulting in poorer crumb firmness and smaller air cells. The viscoelastic properties of the dough are important to ensure consumer acceptance of the product [31].

### 3.5. Accelerated Mould Growth Inhibition Test

Although other studies have been conducted using olive by-products to fortify bakery products, there are no studies that have examined their effect on microbiology. Galanakis et al. [16] used polyphenol extracts from olive mill waste waters, but not the natural matrix, with an antioxidant activity of 142.7 g ABTS g^−1^. As the concentration of the extract increased, a decrease in moulds and yeasts and slower growth were observed as time progressed. With an extract concentration of 200 mg kg^−1^ of olive polyphenols, bread shelf life was increased by 10 to 15 days, reducing the growth of coliforms, *Bacillus* sp., moulds, and yeasts.

There are numerous studies describing the antimicrobial capacity of polyphenols present in olives and their by-products [32,33,34]. In our trial, an inhibitory effect on mould growth was observed throughout the growth period. Other studies have shown that these compounds have effects on the growth of microorganisms and fungi [35,36,37]. The results obtained in our trial may be due to the fact that these compounds are found in low concentrations and, therefore, have a milder, albeit significant, effect on increasing bread shelf life. The increase in temperature due to baking may have affected the concentration of polyphenols, reducing their effect [26,27].

### 3.6. Descriptive Sensorial Analysis

One of the greatest difficulties of fortification is consumer acceptance. Organoleptic characteristics establish the criteria by which consumers judge whether or not to consume a food. A change in organoleptic characteristics can reduce the acceptance of that food, even if the change is not negative [38].

Comparing our different results, we observed that fortification with HQDO in concentrations between 1% and 5% maintained a similar acceptance to CON bread except in “Appearance” and “Texture”. The panellists highlighted that the touch of olive flavour gave it attractive nuances. The darker colouring and a more compact and less light dough distanced its appearance from that of a conventional bread, which may explain this drop in results. However, in no case do the results fall below the acceptability threshold of 5, indicating that these products may be of interest to the consumer. A more extensive study is needed to complement this information.

In the case of Dahdah et al. [14], acceptability remained at 1% incorporation of olive pomace, but at higher concentrations, acceptance decreased. Cedola et al. [12] also observed a decrease in sensory quality when adding olive pomace to bread and pasta, although, in all cases, the acceptability threshold was maintained. Conte et al. [13] also observed a positive response in the sensory analysis of gluten-free breadsticks enriched with phenol-rich extracts from olive oil by-products. Wirkijowska, A., et al. [30] used flaxseed by-products (flour and marc) to fortify breads and observed that even additions of up to 10% exceeded the product’s acceptability threshold. On the other hand, Cecchi et al. [11] obtained more negative results when incorporating olive pâté into bread, pasta, and granola bars. In all cases, the fortified products did not exceed the acceptability threshold, highlighting the strong bitter taste imparted by the olive pâté.

Achieving good sensory properties in fortified products is very important in order to spark consumers’ interest in consuming healthier products with better nutraceutical quality.

## 4. Materials and Methods

### 4.1. Raw Materials

Bread ingredients (strength of flour, EVOO, fresh yeast, white sugar, and table salt) were purchased at a local supermarket (Murcia, Spain). The nutritional composition of the commercial strength flour used is shown in Table 4. The fresh olive pomace by-product (*cv* “Arbequina”) was kindly provided by La Estación S.L. oil mill (Puerto Lumbreras, Murcia, Spain) in December 2024. This fresh pomace was centrifuged (Magnus 21, Ortoalresa, Madrid, Spain; 20 min, 3000 rcf) the same day of collection. The liquid fraction was separated and not used in this study. The centrifuged solid fraction (<50% humidity) was then dried (70 °C, 1 m s^−1^, 5–6% air relative humidity) over 4 h, using a prototype horizontal forced-air dryer with a built-in precision balance (±0.01 g, model AH-300, Gram Precisión S.L.; Barcelona, Spain). Air temperature and humidity were determined using a thermohygrometer (PCE-555, PCE Ibérica S.L.; Albacete, Spain). It was dried until a constant weight was recorded. The solid fraction was then ground (Bosch TSM6A013B, Bosch; Stuttgart, Germany) to obtain the HQDO and stored in hermetically sealed containers, in darkness, and at room temperature until use. Nutritional analysis of HQDO was performed by an external laboratory (Laboratorio Químico Microbiológico S.L., Murcia, Spain) following the methodologies indicated in Table 5.

### 4.2. Bread Making

The recipe used by Cedola et al. [12] was adapted to be made in a bread machine (SilverCrest automatic breadmaker, SBB 850 E1 TARGA^®^ GmbH; Soest, Germany). Three bread recipes with flour partially replaced by HQDO were made to compare with an unfortified control bread (CON). Proportions and ingredients of each bread can be seen in Table 6. The total time for the process was 3 h, divided into kneading (20 min.), fermentation (90 min.), and cooking (70 min.). The baking temperature was 130 °C.

### 4.3. Crust and Crumb Colourimetry

Colour was measured using a colourimeter (Portable Colour Difference Meter TCD100, Beijing TIME High Technology Ltd., Beijing, China). A total of 10 measurements were taken in areas of the bread with fewer irregularities so that the measurement would be as representative as possible, following the method of Giovanelli & Cappa [39]. The measurements obtained are expressed using the CIELab colourimetric system, where L*: luminosity (from 0 to 100, 0 being black and 100 being white), b*: blue/yellow ratio and a*: green/red ratio (from −60 to 60, respectively, in both cases). Total colour difference (TCD), browning index for crust (BI), hº, and whiteness index (WI) for crumb were calculated, as can be seen in Equations (1)–(4):

(1) $$TCD=\sqrt{\sqrt{{\left(L0-L^{*}\right)}^{2}}+{\left(a0-a^{*}\right)}^{2}+{\left(b0-b^{*}\right)}^{2}}$$

(2) $$WI=100-\sqrt{{\left(100-L\right)}^{2}}+a^{2}+b^{2}$$

(3) $$hº=arctan\frac{a^{*}}{b^{*}}$$

(4) $$x=\frac{a^{*}+1.75L^{*}}{5.645L^{*}+a^{*}-3.012b^{*}}$$

(5) $$BI=\frac{100(x-0.31)}{0.17}$$

### 4.4. Height and Weight Loss

The weight of each loaf was monitored over time to determine the tendency of the dough to lose moisture. The measurements were taken for 4 days (day 0 (post oven) and days 1–3). We refer to the difference between the pre-baked and post-baked weight as weight loss due to baking. Weight measurement was achieved by putting the whole bread in a balance (±0.5 g, Gram Precisión S.L.; Barcelona, Spain) and measuring when the weight stabilises. This measurement was carried out to determine the weight loss due to baking. This parameter is expressed as remaining weight (RW), a percentage loss compared to the initial weight of the bread before baking. This method was adapted from Wirkijowska et al. [28].

(6) $$\%\mathrm{RW}=100-(\frac{T0\;weight-T1\;weight}{T0\;weight}*100)$$

Breads were stored at room temperature and RH between 60 and 70% without packaging.

Height was also measured to determine the structural behaviour of the dough in response to moisture loss over time. Height measurement was performed by placing the entire loaf of bread under the calliper and fixing the movable part.

### 4.5. Texture Evolution

For texture measurement, the protocol described in Renoldi et al. [40] was followed. The method used was AACC Method 74–09 [41]. Samples for texture measurement were taken from the centre of the loaf and cube-shaped with an edge size of 25 mm. This texture measurement was carried out for four days to observe the evolution of the hardness over time. Three samples were measured for each day and for each experimental loaf.

### 4.6. Phenolic Compounds and Antioxidant Capacity Analyses

Extracts for the analysis of phenolic compounds and total antioxidant capacity were obtained using ISO 14502-1 methodology, with slight modifications [42]. A bread sample was ground to obtain a fine powder using a manual mortar, and 1 g was accurately weighed (±0.01 g, model AH-300, Gram Precisión S.L.; Barcelona, Spain) to perform the extraction. A total of 100 ml of a water/MeOH mixture (90/10 volume/volume (*v*/*v*)) heated to 60 °C was added to the bread sample previously weighed. The mixture was vigorously shaken for 2 min and left to cool to room temperature for 30 min. The mixture was then filtered using PTFE filters (0.45 micrometres). The filtrate obtained was used as an extract for total phenolic content (TPC) and total antioxidant capacity (TAC)

To analyse the TPC of the above extracts, the method by Singleton & Rossi [43] was used. Briefly, 1 mL of the extract was mixed with 5 mL of Folin–Ciocalteu’s reagent (0.2 N) and left to react for 8 min, after which 4 mL of a 0.7 M sodium carbonate solution was added. Then, the mixture was incubated for 1 h at room temperature in the dark, and finally, the absorbance was measured at 765 nm in a spectrophotometer (Nicolet Evolution 300, Thermo; Waltham, MA, USA). Results were expressed as gallic acid equivalents (GAE) per dry weight in mg g^−1^.

TAC analysis of the above extracts was performed according to the DPPH (2,2-diphenyl-1-picrylhydrazyl) method [44]. Briefly, 1.2 mL of adjusted DPPH solution (adjusted to 1.10 ± 0.02 absorbance at 517 nm) was added to 0.4 mL of the extract and incubated in the dark at room temperature for 10 min. The absorbance at 517 nm was then measured in the spectrophotometer (Nicolet Evolution 300, Thermo; Waltham, MA, USA). The results were expressed as ascorbic acid equivalents (AAEs) per dry weight in mg g^−1^.

### 4.7. Accelerated Mould Growth Inhibition Test

For the accelerated mould control study, each of the four breads was baked on the same day according to the recipe described in Section 2.2. After baking, six slices of each of the breads were cut, and each was placed in a hermetically sealed plastic bag for an accelerated trial. Before that, slices were allowed to cool to room temperature for 30 min. This was completed to avoid excessive condensation inside the bag. Briefly, 5 g of bread was suspended in 50 mL of saline solution (0.8% NaCl) and digested in a stomacher (Panoramic, IUL Instruments, Barcelona, Spain). Appropriate dilutions were made in order to quantify moulds and yeast by using pink bengal + chloramphenicol agar (Microkit labs, Madrid, Spain), in triplicate. Volume of inoculum was 100 µL. Plates were incubated at 27 °C (WTC Binder FD53, Binder GmbH, Tuttlingen, Germany) and counted after three days of incubation.

### 4.8. Descriptive Sensorial Analysis

Bread samples were evaluated with a descriptive sensorial analysis by trained panellists. For panellist training and selection, we followed the protocol of Campo et al. [45]. Twelve candidates were recruited from staff of the Veterinary Faculty and last-year students of the Food Science and Technology at the University of Murcia (n = 12, 6 men and 6 women, ages ranging from 20 to 60) [45]. The sensory analysis was carried out using numbered 10 mm thick samples of each bread recipe. The descriptive analysis questions are items on a rating scale (hedonic from 1 to 10, with 1 being not at all acceptable or not at all intense, 10 being very acceptable or intense, and 5 being the acceptability threshold) and on the subjective perception of panellists on organoleptic characteristics of each sample [46]. This model of questions is referred to as Likert scale questions [47]. The parameters to be analysed in the sensory analysis were as follows:

– Appearance: The following values are included in this attribute: crust thickness, internal colour, external colour, and crumb size.
– Texture: The following values are included in this attribute: crust chewiness and crumb chewiness and astringency.
– Aroma: The following aromas are included in this attribute: toasted, nutty, yeasty, rancid oil, grain, and earthy.
– Flavour: In this case, the evaluation was given according to the intensity with which the tasters detected the different flavours: sweet, salt, sour, bitter, etc.
– Aftertaste: The following flavours are included in this attribute: sweet, sour, astringent, salty, bitter, and olives.

Tests were carried out 3 times, several days apart, for a total of 36 evaluations.

### 4.9. Statistical Analysis

The effect of the addition of HQDO on the different parameters investigated was evaluated by means of a one-way ANOVA for the data sets comparing different types of bread and a two-way ANOVA for the data sets comparing different types of bread at a specific time and the same type of bread at different times. Fisher’s least significant difference (LSD) test was performed to find statistically significant differences (*p* < 0.05). All determinations were made in triplicate, and data were expressed as mean ± SD. The tests were performed using STATGRAPHICS Centurion v15.2 software (2025 Statgraphics Technologies, Inc.; The Plains, VA, USA).

## 5. Conclusions

The impact of adding HQDO on the nutraceutical and sensory characteristics of bread was evaluated. The results showed that fortifying bread with HQDO improved its nutraceutical quality, increasing significantly both the phenolic content (0.19 mg GAE g^−1^ DW in unfortified bread to 0.73 mg GAE g^−1^ DW at 10% fortification (*p* < 0.05)) and antioxidant activity (from 1.24 mg AAE g^−1^ DW in unfortified bread to 1.49 mg AAE g^−1^ DW at 10% fortification (*p* < 0.05)).

In terms of sensory properties, the values obtained decrease as HQDO incorporation increases. However, all bread samples fortified with HDQO showed good sensory qualities, with values close to seven or higher. The most drastic changes were in “Appearance” (9.1 CON to 7.3 at 10% fortification (*p* < 0.05)) and “Texture” (9.3 CON to 7.3 at 10% fortification (*p* < 0.05)). In the case of “Flavour” (8.1 CON to 6.7 at 10% fortification (*p* < 0.05)) and “Aftertaste” (7.7 CON to 6.8 at 10% fortification (*p* < 0.05)), the change was minor. It should be noted that “Aroma” was the least affected parameter (7.5 CON to 7.1 at 10% fortification) and even obtained a higher value at 1% fortification of 8.5.

These results open up new possibilities for incorporating HDQO as a new ingredient in flour for bread and bakery products. The new HDQO-fortified breads offer good nutritional and nutraceutical characteristics due to their concentration of phenolic compounds, antioxidant capacity, dietary fibre, and unsaturated fats. The results of this study may benefit both the olive oil industry in reducing its environmental impact and by-product valorisation and the flour industry in developing new formulations with HDQO to produce new healthy foods.

## Figures and Tables

**Table 1:** Crust colourimetry results.

Sample	L*	a*	b*	BI	hº	TCD
CON	64.3 ± 1.1 ^a^	5.1 ± 0.2 ^b^	24.0 ± 0.4 ^a^	51.7 ± 1.1 ^b^	1.4 ± 0.0 ^a^	
HQDO1	53.5 ± 7.7 ^b^	9.0 ± 4.2 ^b^	22.0 ± 4.1 ^a^	64.8 ± 7.7 ^ab^	1.2 ± 0.1 ^b^	12.7 ± 7.4 ^b^
HQDO5	43.5 ± 4.3 ^c^	9.5 ± 0.8 ^b^	16.9 ± 0.7 ^b^	67.2 ± 4.3 ^ab^	1.0 ± 0.0 ^bc^	22.5 ± 4.1 ^b^
HQDO10	30.6 ± 3.2 ^d^	8.7 ± 1.2 ^a^	13.9 ± 2.5 ^b^	81.6 ± 3.1 ^a^	1.0 ± 0.1 ^c^	35.5 ± 3.0 ^a^

In CIELab system, L* indicates lightness, a* is the red/green coordinate, and b* is the yellow/blue coordinate. In CIELab system, hº represents he hue of a color in degrees. Means ± standard deviation values followed by different letters in the same column are different (*p* < 0.05). Each variable that shares the same letter (a, b, c, and d) shares a mean that is not statistically different from another.

**Table 2:** Crumb colourimetry results of the four bread samples.

Sample	L*	a*	b*	WI	hº	TCD
CON	56.0 ± 1.8 ^a^	1.9 ± 1.3 ^c^	12.7 ± 2.0 ^a^	54.1 ± 1.4 ^a^	1.4 ± 0.1 ^a^	
HQDO1	43.8 ± 8.2 ^b^	3.9 ± 1.5 ^bc^	13.0 ± 0.6 ^a^	42.1 ± 8.2 ^b^	1.3 ± 0.1 ^a^	12.4 ± 8.4 ^b^
HQDO5	31.6 ± 6.7 ^c^	5.9 ± 1.4 ^ab^	9.4 ± 3.1 ^a^	30.6 ± 6.3 ^c^	1.0 ± 0.2 ^b^	25.1 ± 6.8 ^ab^
HQDO10	28.7 ± 3.5 ^c^	8.1 ± 1.6 ^a^	9.3 ± 1.5 ^a^	27.6 ± 3.2 ^c^	0.8 ± 0.1 ^b^	28.3 ± 3.4 ^a^

In CIELab system, L* indicates lightness, a* is the red/green coordinate, and b* is the yellow/blue coordinate. In CIELab system, hº represents he hue of a color in degrees. Means ± standard deviation values followed by different letters in the same column are different (*p* < 0.05). Each variable that shares the same letter (a, b, ab, bc, and c) shares a mean that is not statistically different from another.

**Table 3:** Sensory evaluation of the four bread samples.

Sample	Appearance	Texture	Aroma	Flavour	Aftertaste
CON	9.1 ± 0.7 ^a^	9.3 ± 0.7 ^a^	7.5 ± 1.2 ^b^	8.1 ± 1.2 ^a^	7.7 ± 1.6 ^a^
HQDO1	7.5 ± 2.2 ^b^	7.9 ± 2.3 ^b^	8.5 ± 1.0 ^a^	7.9 ± 1.7 ^a^	7.6 ± 1.7 ^a^
HQDO5	7.6 ± 1.1 ^b^	7.2 ± 1.3 ^c^	7.5 ± 1.6 ^b^	7.9 ± 1.2 ^a^	8.0 ± 0.7 ^a^
HQDO10	7.3 ± 1.4 ^b^	7.3 ± 1.2 ^bc^	7.1 ± 1.1 ^b^	6.7 ± 1.6 ^b^	6.8 ± 1.4 ^b^

Means ± standard deviation values followed by different letters in the same column are different (*p* < 0.05). Each variable that shares the same letter (a, b, bc, and c) shares a mean that is not statistically different from another.

**Table 4:** Nutritional composition of commercial strength flour.

Fractions	Weight (g 100 g^−1^)
Total fats	0.9
Saturated fats	0.2
Total carbohydrates	68.8
Sugars	0.4
Dietary fibre	2.5

**Table 5:** Nutritional composition of HQDO.

Fractions	Weight (g 100 g^−1^)	Method
Total fats	17.2 ± 1.7	§64 LFBG L 17.00-4, mod.:2017-10, Weibull-Stoldt-Gravimetry
Saturated fats	2.7 ± 0.27	DGF C-VI 10a:2023
Total carbohydrates	17.4 ± 6.1	§64 LFBG L 17.00-4, mod.:2017-10
Sugars	8.8 ± 2.6	§64 LFBG L 17.00-4, mod.:2017-10
Dietary fibre	48.0 ± 17	§64 LFGB L 00.00-18: 1997-01
Proteins	7.6 ± 0.76	§64 LFBG L 17.00-15:2013-08, Kjeldahl
Salt	0.015 ± 0.0023	§64 LFBG L 17.00-4, mod.:2017-10

**Table 6:** Composition of breads.

Sample ^1^	Flour (g)	Water (ml)	HQDO (g)	Yeast (g)	Sugar (g)	Salt (g)	Olive Oil (g)
CON	500	300	0	15	5	10	20
HQDO1	495	300	5	15	5	10	20
HQDO5	475	300	25	15	5	10	20
HQDO10	450	300	50	15	5	10	20

^1^ CON: unfortified bread (control); HQDO1: 1% flour substitution; HQDO5: 5% flour substitution; HQDO10: 10% flour substitution.

## Data Availability

The data presented in this study are available upon request from the corresponding author.
